# Imaging Tool for Predicting Renal Clear Cell Carcinoma Fuhrman Grade: Comparing R.E.N.A.L. Nephrometry Score and CT Texture Analysis

**DOI:** 10.1155/2021/1821876

**Published:** 2021-12-23

**Authors:** Ran Sun, Sheng Zhao, Huijie Jiang, Hao Jiang, Yanmei Dai, Chuzhen Zhang, Song Wang

**Affiliations:** ^1^Department of Radiology, The Second Affiliated Hospital of Harbin Medical University, Harbin, China; ^2^Department of Radiology, Longhua Hospital, Shanghai University of Traditional Chinese Medicine, China

## Abstract

**Background:**

Clear cell renal cell carcinoma (ccRCC) is the most common renal malignant tumor. Preoperative imaging boasts advantages in diagnosing and choosing treatment methods for ccRCC.

**Purpose:**

This study is aimed at building models based on R.E.N.A.L. nephrometry score (RNS) and CT texture analysis (CTTA) to estimate the Fuhrman grade of ccRCC and comparing the advantages and disadvantages of the two models.

**Materials and Methods:**

143 patients with pathologically confirmed ccRCC were enrolled. All patients were stratified into Fuhrman low-grade and high-grade groups with complete CT data and R.E.N.A.L. nephrometry scores. CTTA features were extracted from the ROI delineated at the largest tumor level, and RNS and CTTA features were included in the logistic regression model, respectively.

**Results:**

RNS model constructed based on multivariate logistic regression analysis showed that 3 pts for *R*-scores, 2 pts for *E*-scores, and 3 pts for *L*-scores were significant indicators to predict high-grade ccRCC, the AUC of RNS model was 0.911, and the sensitivity and specificity were 71.11% and 83.67%, respectively. The CTTA-model confirmed energy, kurtosis, and entropy as independent predictive factors, and the AUC of CTTA model was 0.941, with an optimal sensitivity and specificity of 84.44% and 93.88%.

**Conclusions:**

R.E.N.A.L. nephrometry score has a certain provocative effect on the Fuhrman pathological grading of ccRCC. As a potential emerging technology, CTTA is expected to replace R.E.N.A.L. nephrometry score in evaluating patients' Fuhrman classification, and this approach might become an available method for assisting clinicians in choosing appropriate operation.

## 1. Introduction

Renal cell carcinoma (RCC) is the most common renal malignant tumor, originating from the renal parenchymal urinary epithelial system and accounting for 80%–90% of primary renal malignant tumors. Recently, a global report about RCC has suggested that the morbidity and mortality rates of RCC are increasing by year [1]. As compared with other subtypes of RCC, clear cell renal cell carcinoma (ccRCC) is the most common subtype of RCC, which accounts for 70%-80% of RCC [1, 2].

Prognostic assessment of cancers is one of core clinical mission, especially to ccRCC, which is a heterogeneous disease with poor prognosis [3]; tumor grading is a key prognostic factor with the increasing appreciation [4]. Fuhrman classification scheme was widely used in the pathological grading of RCC and was one of the main factors suggesting affecting the prognosis of RCC. This pathological grading system was originally proposed by Fuhrman et al. [5], who mainly classified tumor cells into four grades based on the size of the nucleus and the morphology of the nucleoli. Previous studies identified the prognosis of Fuhrman low-grade (Fuhrman grades I or II) ccRCC patients is relatively better than Fuhrman high-grade patients, the safety of partial nephrectomy is higher, and the incidence of postoperative complications is lower [6]. However, in high-grade (Fuhrman grades III or IV) ccRCC patients, the situation is reversed, and the appropriate treatment options available are different. Many studies have shown that the traditional four-grade classification system can be simplified into a two-grade system of a low-grade group and high-grade group [7, 8]. With this new approach, the accuracy of the evaluation method is unchanged, but the consistency is higher. However, since Fuhrman classification is a pathological grading standard usually only be obtained through postoperative pathology, how we can obtain the patients' Fuhrman classification in a timely and effective manner before treatment is still a problem that we need to address.

As a common way to gather clinical evidence, preoperative imaging plays a decisive role in the diagnosis and clinical treatment of RCC. At present, clinicians and researchers have proposed more than 10-related scoring methods in the literature, the first-generation scoring system, including R.E.N.A.L. nephrometry score (RNS), the PADUA scoring system, and the C-index (CI) system are widely used [9]. RNS was first proposed by Kutikov and Uzzo [10] and is a preoperative anatomical evaluation method for assessing renal tumors based on image data. The indicators are relatively objective, and the measurement stability is strong and robust. Five scoring items compose this system and can objectively evaluate the surgical difficulty of renal tumors. Lesions with a final score of four to six points (pts) are of a low complexity, tumors of seven to nine pts are of moderate complexity, and those of 10 to 12 pts are considered to be highly complex; based on the score, clinicians can choose the most reasonable surgical method and treatment in each case [6].

On the other hand, as a “radiomics” method for medical image interpretation, CT texture analysis (CTTA) can effectively describe the spatial distribution of image gray intensity. It has become a promising technology for evaluating tumor heterogeneity and predicting treatment response and prognosis. The image information obtained by texture analysis cannot be recognized by naked eyes [11, 12]. In CTTA, the feature that quantitatively describes the distribution of pixel signal intensity in the target area is called first-order texture feature, also known as histogram feature. Previous studies have shown that the first-order texture feature of CCTA is related to clear cell histologic findings of RCC and can predict the time of disease recurrence and death due to disease [13]. Studies have shown that CT or MRI texture analysis can distinguish between ccRCC and non-ccRCC based on the extracted texture features [14, 15]. Additionally, some studies have exhibited that texture parameters such as entropy are related to the Fuhrman grade of RCC and TNM stage [14, 16, 17]. Thus, this study is aimed at building models based on R.E.N.A.L. nephrometry score and CTTA, sought to estimate the Fuhrman grade of ccRCC by the two models, and comparing the pros and cons of the two methods.

## 2. Materials and Methods

### 2.1. Patient Population

**Figure 1:**
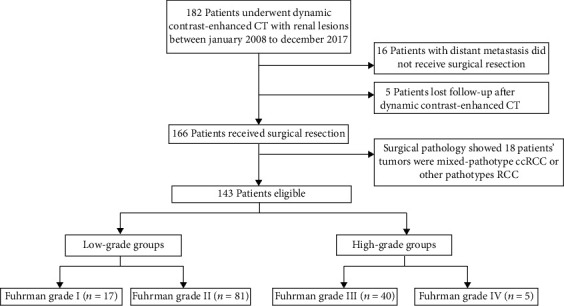
Patient selection process.

This study was a single-center retrospective study and approved by the Medical Ethics Committee of the Second Affiliated Hospital of Harbin Medical University, and due to the retrospective character of this study, the informed consent was waived. From January 2008 to December 2017, this study collected a total of 182 consecutive patients suspected ccRCC by dynamic contrast-enhanced CT at our hospital. Cases were included if meeting the following criteria: (1) complete preoperative dynamic contrast-enhanced CT images, including cross-sectional image slices and coronal and sagittal MRPs, clearly showed lesion boundaries; (2) patients were confirmed surgical pathology with single-pathotype ccRCC, and the location of tumors was unilateral and solitary; (3) without distant metastasis or renal dysplasia. A total of 39 patients were excluded, including 16 patients with other organ or tissue metastasis, five patients lost follow-up after dynamic contrast-enhanced CT, and surgical pathology showed 18 patients' tumors were mixed-pathotype ccRCC or other pathotypes RCC. Finally, according to the 7th Edition of the AJCC cancer staging manual, we stratified Fuhrman grade I and II as low-grade groups (LGG) and Fuhrman grade III and III as high-grade group (HGG).  Figure 1 presents the patient recruitment process.

### 2.2. CT Scanning Protocol

Routine dynamic CT was performed on a dual source multidetector (64-section) row Definition Flash® CT scanner (Siemens, Munich, Germany). All patients fasted 8 hours and drank about 1 L of water in 30 minutes before scanning and scanned abdomen, in the supine position injecting nonionic contrast material (Iohexol, 400 mg/dl Iodine, calculated as 1.5 ml per kilogram of body weight) at the rate of 4 ml/s pumped through a double-barrel high-pressure syringe (Oelrich, Germany) [18]. The scanning protocol began about 5 seconds after the injection of the contrast agent. The scan time in the corticomedullary phase was 25 seconds and that in the nephrographic phase was 60 seconds; 120 seconds later, the excretory phase scan was initiated. The scanning parameters were the following, slice thickness 5 mm, layer spacing 5.0 mm, pitch 1.2, reconstruction interval 1.25 mm, tube current 300 mA, and tube voltage 120 kV.

### 2.3. Image Analysis

**Figure 2:**
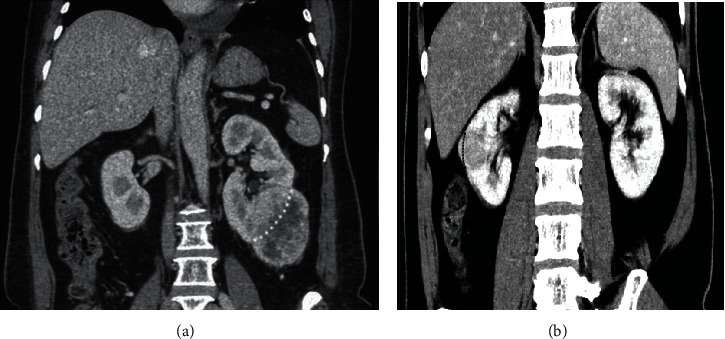
(a) shows a left kidney tumor (*E* − scores = 1), with the white dotted kidney outline as the boundary, where the tumor is mostly located outside the kidney. (b) presents a right kidney tumor *E* − scores = 2, with the black dotted kidney outline as the boundary, where the tumor is mostly located inside the kidney but a small part is still located outside the kidney.

**Figure 3:**
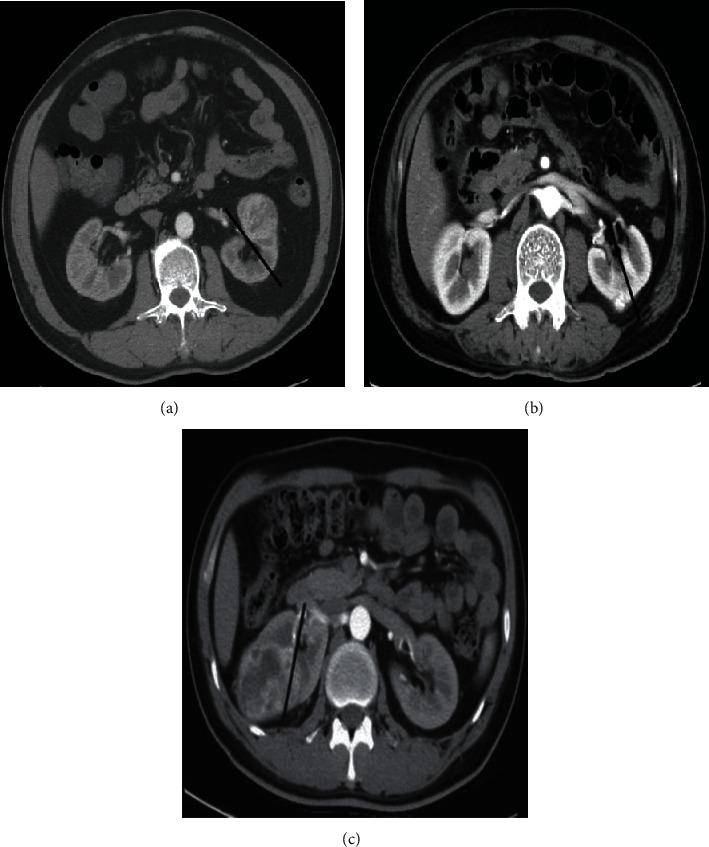
The solid line is parallel to the anterior and posterior lip centerline and divides the renal parenchyma into the ventral and dorsal sides. (a) shows the left kidney tumor on the ventral side *A*-scores; (b) shows the left kidney tumor on the dorsal side (*P*), and (c) shows the right kidney tumor spanning the midline and where it cannot be located.

**Figure 4:**
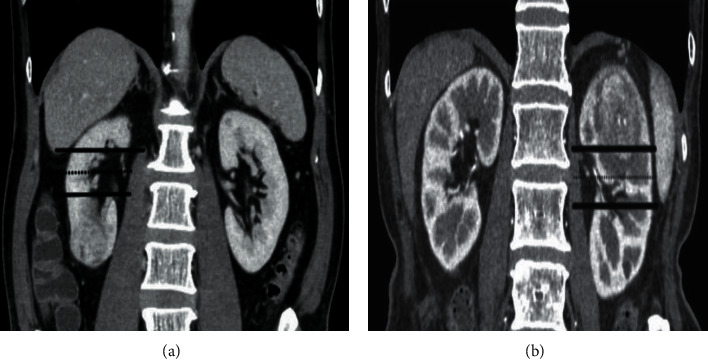
The dotted line is parallel to the midline of the upper and lower lips of the kidney and the solid line is the upper and lower polar lines of the kidney. (a) shows that the right kidney tumor is completely located at the lower pole, with *L* − scores = 1. (b) shows that the left kidney tumor crosses the upper pole of the renal line but does not cross the midline of the kidney and remains mostly still at the upper pole, *L* − scores = 2; meanwhile, another tumor is partially located between the upper and lower polar lines, *L* − scores = 3.

The R.E.N.A.L. nephrometry scores on CT were determined subjectively by a radiologist with 10 years of experience in abdominal imaging, based on the R.E.N.A.L. nephrometry score standard [10]. Whether each patient has tumor blood vessels, select the lesion with the largest diameter. The images of all included cases were evaluated according to Table 1. As shown in Figures 2–4, the maximum diameter of the tumor *R*-score, *E*-scores (the convexity of the tumor), *N*-scores (the positioning of the tumor relative to the renal collecting system or sinus), *A*-scores (measured by the distance between the medial side of the tumor and the renal system or renal sinus fat the position of the tumor in the axial position), and *L*-scores (the relationship between the tumor and the renal polar line). The *R*-score, *E*-score, and *N*-scores indicators in particular needed to be comprehensively evaluated in the axial, coronary, and sagittal positions. Moreover, imaging features such as necrosis and calcification in the lesions were observed and recorded. All data were measured triple at different times by the radiologist and averaged.

### 2.4. CT Texture Feature Extraction

The digital imaging and communication (DICOM) files of included patients were exported by the image archiving and communication system (PACS) and anonymized. The images were read using the open-source imaging postprocessing software 3D slicer software (version 4.11.20210226), and a radiologist with 20 years of experience in abdominal imaging (HJJ) draws a region of interest (ROI) along the edge of the tumor on the largest level of the axial tumor and blinded to pathology. The texture features were extracted by a 3D slicer extension (SlicerRadiomics). Based on the ROI, 18 first-order features were obtained. In order to reduce the impact of texture analysis feature collinearity on the subsequent model construction, we use Spearman rank correlation to remove the features with high correlation (coefficient ∣*ρ* | ≥0.9).

### 2.5. Statistical Analysis

The Statistical Package for the Social Sciences (SPSS) version 20.0 software program (IBM Corp., Armonk, NY, USA) and R software (version 4.0.4, https://www.r-project.org/) was used for statistical analysis. Measurement data were expressed as *x̅*±*s*. The chi-squared test was used to compare the differences in RNS and gender between the Fuhrman high- and low-grade groups. The student's *t*-test was performed to assess the differences in age and every item of RNS between the high-grade and low-grade groups. Multivariate logistic regression analysis was used to distinguish two groups with respect to statistical significance of texture features and a scoring item. The receiver operating characteristic (ROC) curve was drawn to compare multiparameter regression models, scores, and the diagnostic value of each single scoring item and to assess sensitivity and specificity of the total scoring item by threshold analysis. DeLong testing was used to compare area under the curve (AUC) between RNS and texture analysis models. The statistical methods used were selected according to the “Guidelines for reporting of statistics for clinical research in urology” [19]. Statistical results were statistically significant according to *p* < 0.05.

## 3. Results

### 3.1. Clinical and Pathological Characteristics of Patients

Finally, 143 patients were enrolled in the study, and the mean age was 58.4 years in the low-grade group and 60.3 year in the high-grade group. Males had a preponderance in both groups (63.3% and 73.3%, respectively). A summary of patient demographics, T stage, and tumor distribution is provided in Table 1. Age, gender, necrosis, calcification, T stage, and tumor distribution showed no significant difference between the low-grade group and the high-grade group (*p* > 0.05) Table 2.

### 3.2. R.E.N.A.L. Nephrometry Score Evaluating Fuhrman Classification

We calculated R.E.N.A.L. nephrometry score of the tumors between the low-grade group and the high-grade group and summarized them in Table 3. The *R*-scores, *E*-scores, and *N*-scores of tumors in the high-grade group were relatively lower than the low-grade group (*p* < 0.05), but *L*-scores and *A*-scores had no significant difference between the two groups (*p* = 0.135 and 0.104, respectively) Table 4. Compared with Fuhrman low-grade, the total RNS items (3 pts for *R*-scores, 2 pts for *E*-scores, and 3 pts for *L*-scores) were associated with Fuhrman high-grade, and the difference was statistically significant (*p* < 0.001). RNS model constructed based on multivariate logistic regression analysis showed that 3 pts for *R*-scores (largest tumor diameter), 2 pts for *E*-scores (tumor convexity), and 3 pts for *L*-scores (relationship between tumor and renal polar line) were significant indicators to predict high-grade ccRCC.

### 3.3. CTTA Model Construction

**Figure 5:**
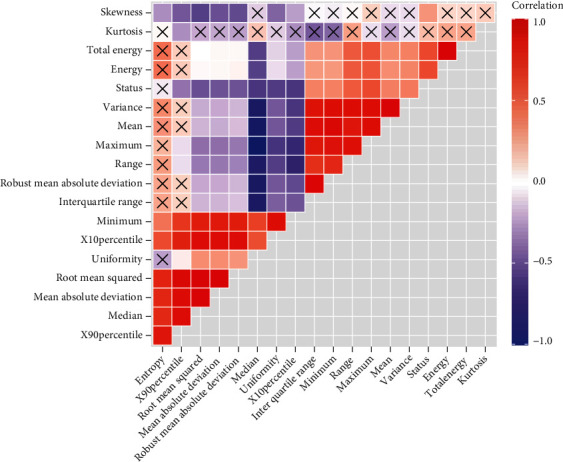
The correlation coefficient heat map of texture features. The heat map shows the correlation coefficients of all 18 texture features. The red square indicates a positive correlation, and the purple square indicates a negative correlation. The correlation degree is consistent with the color depth, and the cross indicates that there is no correlation between the two features.

In the 18 extracted texture features, 7 texture features with high collinearity were removed (coefficient ∣*ρ* | ≥0.9), and the remaining 11 features were input into the logistic regression via backward stepwise model (Figure 5). The CTTA-model confirmed energy (OR = 1.000, 95% CI 0.001-1167.084, *p* < 0.001), kurtosis (OR = 0.438, 95% CI: 0.008-23.914, *p* = 0.041), and entropy (OR = 1.506, 95% CI 0.015-65.30, *p* = 0.033) as independent predictive factors, and the AUC of CTTA-model was 0.941, with an optimal sensitivity and specificity of 84.44% and 93.88%.

### 3.4. Performance and Validation of the R.E.N.A.L. Nephrometry Score and CTTA

The AUCs of *R*-scores, *E*-scores, *L*-scores, and *N*-scores were 0.79, 0.78, 0.73, and 0.60, respectively (Figures 4(a) and 4(b)). Delong test showed that the AUC of RNS model and the total RNS items (0.909 and 0.786, respectively) was larger than the AUC of each single RNS score, and the differences were statistically significant (*p* < 0.05). There was no significant difference in ROC curves between RNS model and CTTA-model (*p* = 0.783).

The calibration curves (Figures 5(c) and 5(d)) showed the matching degree between the prediction probability and the actual probability of CTTA model and RNS model and indicated that CTTA-model had better consistency between observation and prediction.

## 4. Discussion

**Figure 6:**
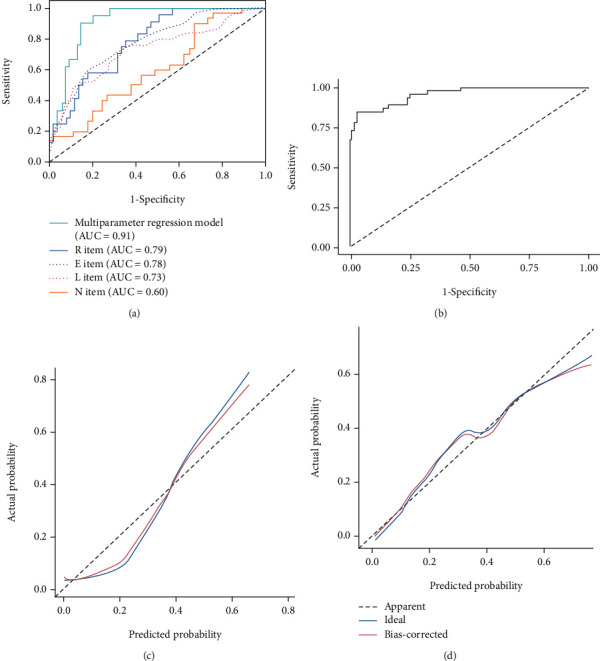
ROC curves of R.E.N.A.L. nephrometry score and CT texture analysis models in differentiating high-grade ccRCC groups from low-grade ccRCC groups. (a) ROC curves of RNS model and R.E.N.A.L. score items. (b) ROC curve of texture analysis model. Then, the calibration curves of R.E.N.A.L. nephrometry score and texture analysis models were used to predict Fuhrman grade in ccRCC patients. The horizontal axis represents the prediction probability, and the vertical axis represents the actual probability. (c) RNS model. (d) CT texture analysis model.

In RCC studies, conventional medical imaging techniques, such as CT, MRI, and PET/CT, had been applied to RCC classification and pretreatment staging. As one of the conventional morphological imaging evaluation criteria, previous studies demonstrated that R.E.N.A.L. (Figure 6) nephrometry score can be used to identify high-risk and low-risk renal tumors, predict RCC postoperative recurrence, assess preoperative stage, and evaluate tumor proliferation activity [20–23]. In contrast, radiomics and texture analysis started to receive increasing attention as a new approach that differs from conventional medical imaging assessment methods. Radiomics and texture analysis involved medical imaging extraction of quantitative features and provided a novel idea for tumor preoperative evaluation by correlating the imaging features with the patient's clinicopathological characteristics [24]. Fuhrman classification is the most used pathological grading system for RCC and is also one of the critical independent factors suggesting the prognosis of RCC. Hence, determining patients' Fuhrman classification before surgery has vital clinical significance in the treatment and prognosis of ccRCC patients. Interestingly, current studies stated that R.E.N.A.L. nephrometry score and CT texture analysis can be used to identify Fuhrman high-grade and low-grade ccRCC [14, 25]. Our research aimed to apply these two methods for evaluating the Fuhrman classification of ccRCC and comparing the advantages and disadvantages of them.

Tumor size is an important part of the TNM staging of RCC and is also currently the only effective indicator for monitoring tumor progression [26]. In this work, the results of each single score item indicated that the difference in tumor size (*R*-score) between the high-grade and low-grade ccRCC groups was statistically significant. Moreover, R.E.N.A.L. nephrometry score was relatively higher in high-grade ccRCC group. Importantly, multivariate logistic regression analysis illustrated that, as compared within one pt for *R*-score, three pts for *R*-score displayed more significant indicators concerning predicting high-grade ccRCC. Previous study has shown that the combination of tumor size and patient symptoms can accurately stratify the survival of RCC patients. This result implied that tumor size is related to Fuhrman classification, and the larger the lesion, the greater the risk of high-grade tumors [27]. Similarly, Jeldres et al. analysed a large amount of data and found that 91.5% of renal masses less than or equal to 2 cm did not contain Fuhrman high-grade pathological components [28].

**Figure 7:**
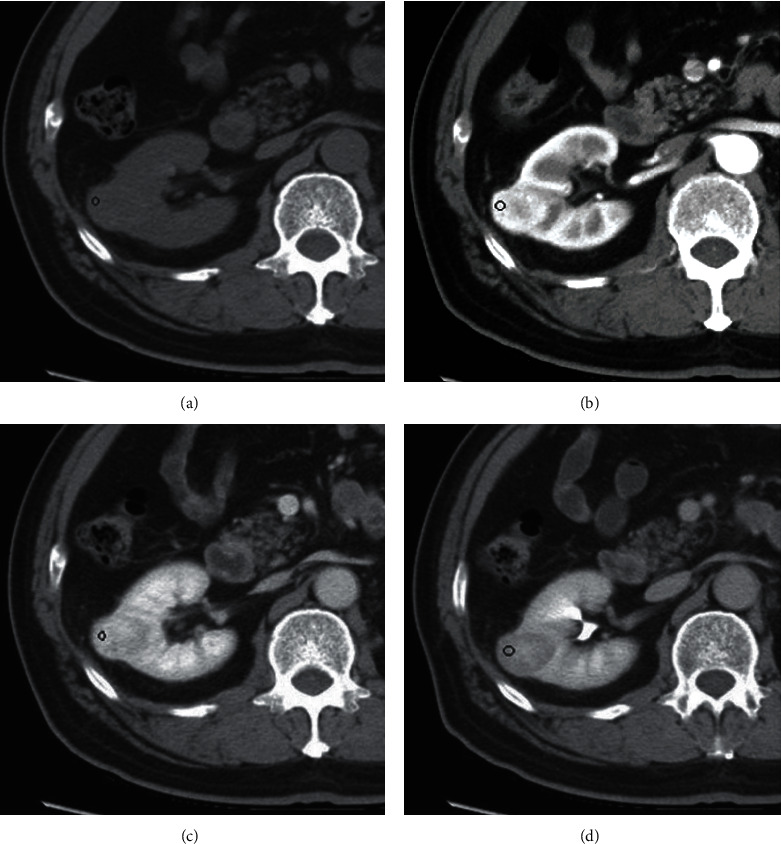
A 75-year-old man with postoperative pathologically confirmed ccRCC in the right middle kidney. A region of interest (ROI) was set in the corticomedullary phase of the tumor with the most significant enhancement possible. The remaining sections were scanned at the same level as that set for the ROI. (a) Precontrast images, (b) corticomedullary phase, (c) nephrographic phase, and (d) excretory phase.

In the RNS model, the *E*-, *N*-, and *L*-scores were also statistically different between high-grade and low-grade groups, and 2 pts for *E*-scores and 3 pts for *L*-scores showed independent predictors of high-grade ccRCC. Shim et al. showed that lesions located adjacent to the renal arterial trunk and/or vein and branches of the renal hilum often display a higher pathological grade [29]. In our work, the *N*- and *L*-scores of high-grade ccRCC were also higher than low-grade, and the lesions of high-grade ccRCC were positioned closer to the renal hilum, consistent with the conclusions of above. Therefore, tumors located near the sinus in the middle of the kidney are more likely to demonstrate high-grade, but the axial position (ventral/dorsal) of the tumor was not statistically significant (Figure 7).

In addition, some studies have reported that the multivariate regression model with multiple scoring items in the comprehensive R.E.N.A.L. nephrometry score displayed a higher degree of diagnostic efficacy for Fuhrman grading [25]. In our study, the multivariate regression model combining *R*-, *E*-, *N*-, and *L*-scores in this study found the AUC of total score item was better than the AUCs of single score item. Therefore, in predicting Fuhrman's pathological grade using R.E.N.A.L. nephrometry score, it is recommended to use the comprehensive score other than simply a score for further evaluation.

On the other hand, CTTA features have a statistically significant correlation with Fuhrman classification in this study. Deng et al. [14] found the entropy value with fine and medium spatial filters increased significantly in ccRCC. In our study, high entropy was correlated with high Fuhrman grade as expected (*p* = 0.033), and multivariate logistic regression analysis further confirmed the relationship between texture features and Fuhrman grade. Furthermore, some studies have shown that in addition to higher entropy, higher standard deviation, higher kurtosis, and positive skewness are also considered to represent an increase in intratumoral heterogeneity, indicating a worse prognosis [30, 31], the results were also consistent with our study. Nevertheless, the above studies on RCC texture analysis only took single optimal feature as predictive parameter, instead of modeling all optimal features to enhance the diagnostic efficiency, which leads to the limitations of application. In our research, all optimal texture features are used to build models, which improve the diagnostic efficiency and have better application prospects. Since the Delong test showed that there was no statistical difference between the RNS model and CTTA model in the diagnostic efficiency, and their AUCs were both greater than 0.9.Therefore, this study did not attempt to merge the two models. The calibration curve shows that texture analysis model can more accurately predict ccRCC patients' Fuhrman classification. Moreover, texture analysis digitized patient images through data mining, which can effectively eliminate subjective errors that may be caused by R.E.N.A.L. nephrometry score and can make the diagnosis more objective and accurate.

We were aware of limitations of our study. First of all, this study was a single-center retrospective study. The lack of multicenter verification and sample size limitations may hinder the universality of the research results. Second, this study had only one reviewer to evaluate CT images and perform CTTA delineation. However, previous studies have shown that CTTA features had a robust interobserver agreement. Comparing RNS model and texture analysis model, we can find that they have similar diagnostic power, also means that CTTA features with high objective not only have good interpretability but also hope to free radiologists from mechanical tumor CT evaluation.

## 5. Conclusions

To sum up, R.E.N.A.L. nephrometry score has a certain provocative effect on the Fuhrman pathological grading of ccRCC. As a potential emerging technology, CTTA is expected to replace R.E.N.A.L. nephrometry score in evaluating patients' Fuhrman classification, and this approach might become an available method for assisting clinicians in choosing appropriate operation.

## Figures and Tables

**Table 1:** Clinical and pathological characteristics of patients.

Variables	Low-grade group^1^	High-grade group^1^	*p* value^2^
Age	58.4 ± 14.5	60.3 ± 13.4	0.442
Gender			
Male	62 (63.3%)	33 (73.3%)	0.236
Female	36 (36.7%)	12 (26.7%)	
Necrosis			
Yes	76 (77.6%)	41 (91.1%)	0.051
No	22 (22.4%)	4 (8.9%)	
Calcification			
Yes	11 (11.2%)	8 (17.8%)	0.284
No	87 (88.8%)	37 (82.2%)	
T stage			
pT1a	21 (21.43%)	13 (28.89%)	0.510
pT1b	53 (54.08%)	24 (53.33%)	
pT2a	24 (24.49%)	8 (17.78%)	
Kidney			1.000
Left	42 (42.86%)	20 (44.44%)	
Right	56 (57.14%)	25 (55.56%)	

^1^ *n* (%). ^2^Pearson's Chi-squared test; Fisher's exact test.

**Table 2:** R.E.N.A.L. nephrometry score standard.

Index	Score		
	1 pt	2 pts	3 pts
*R* (cm)	≤4	7 > *R* > 4	≥7
*E*	≥50%	<50%	Completely endogenous
*N* (mm)	≥7	7 > *E* > 4	≤4
*A*	*A* (ventral)	*P* (back side)	*X* (unable to determine)
*L*	The tumor is located completely above or below the kidney	Most tumors are in the superior or inferior pole of the kidney	More than 50% of tumors pass through the upper/lower polar line of the kidney or are completely between the renal upper/lower polar line

**Table 3:** Comparing R.E.N.A.L. nephrometry score between the Fuhrman low-grade and high-grade groups.

Variables	High-grade group_1_	Low-grade group^1^	*p* value^2^
*R*-scores			<0.001
1 pt	12 (26.67%)	51 (52.04%)	
2 pts	27 (60.00%)	23 (23.47%)	
3 pts	6 (13.33%)	24 (24.49%)	
*E*-scores			<0.001
1 pt	3 (6.67%)	36 (36.73%)	
2 pts	24 (53.33%)	46 (46.94%)	
3 pts	18 (40.00%)	16 (16.33%)	
*N*-scores			0.030
1 pt	9 (20.00%)	42 (42.86%)	
2 pts	16 (35.56%)	25 (25.51%)	
3 pts	20 (44.44%)	31 (31.63%)	
*A*-scores			0.106
1 pt	21 (46.67%)	29 (29.59%)	
2 pts	19 (42.22%)	49 (50.00%)	
3 pts	5 (11.11%)	20 (20.41%)	
*L*-scores			0.687
1 pt	9 (20.00%)	18 (18.37%)	
2 pts	20 (44.44%)	51 (52.04%)	
3 pts	16 (35.56%)	29 (29.59%)	
1 pt for (*R*), (*E*), and (*L*)	53 (54.1%)	8 (17.7%)	<0.001
3 pts for (*R*) and (*E*), 2 pts for (*L*)	19 (19.4%)	25 (55.6%)	

^1^ *n* (%). ^2^Pearson's Chi-squared test; Fisher's exact test.

**Table 4:** Multivariate logistic regression analysis of R.E.N.A.L. nephrometry score between the Fuhrman low-grade and high-grade groups.

Variables	*B*	*P*	OR	95% CI
*R*-scores (1 pt)	—	—	—	—
*R*-scores (2 pts)	2.12	0.004	8.36	1.94-36.06
*R*-scores (3 pts)	4.31	<0.001	74.52	10.89-509.85
*E*-scores (1 pt)	—	—	—	—
*E*-scores (2 pts)	2.33	<0.001	10.28	2.89-36.48
*E*-scores (3 pts)	1.32	0.397	3.74	0.20-70.36
*N*-scores (1 pts)	—	—	—	—
*N*-scores (2 pts)	0.123	0.909	1.13	0.14-9.43
*N*-scores (3 pts)	-0.097	0.921	0.91	0.14-6.11
*L*-scores (1 pt)	—	—	—	—
*L*-scores (2 pts)	0.854	0.172	2.35	0.69-8.01
*L*-scores (3 pts)	1.36	0.023	3.89	1.21-12.52

## Data Availability

The datasets used and/or analysed during the current study are available from the corresponding authors on reasonable request.
